# Plasticity of monocytes and macrophages in cirrhosis of the liver

**DOI:** 10.3389/fnetp.2022.937739

**Published:** 2022-07-15

**Authors:** Anne Geng, Emilio Flint, Christine Bernsmeier

**Affiliations:** ^1^ Translational Hepatology, Department of Biomedicine, University of Basel, Basel, Switzerland; ^2^ Department of Biomedicine, University of Basel and University Centre for Gastrointestinal and Liver Diseases, Basel, Switzerland

**Keywords:** cirrhosis, monocytes, macrophages, liver injury, inflammation, ACLF, immuneparesis, immunotherapy

## Abstract

Cirrhosis of the liver is a systemic condition with raising prevalence worldwide. Patients with cirrhosis are highly susceptible to develop bacterial infections leading to acute decompensation and acute-on-chronic liver failure both associated with a high morbidity and mortality and sparse therapeutic options other than transplantation. Mononuclear phagocytes play a central role in innate immune responses and represent a first line of defence against pathogens. Their function includes phagocytosis, killing of bacteria, antigen presentation, cytokine production as well as recruitment and activation of immune effector cells. Liver injury and development of cirrhosis induces activation of liver resident Kupffer cells and recruitment of monocytes to the liver. Damage- and pathogen-associated molecular patterns promote systemic inflammation which involves multiple compartments besides the liver, such as the circulation, gut, peritoneal cavity and others. The function of circulating monocytes and tissue macrophages is severely impaired and worsens along with cirrhosis progression. The underlying mechanisms are complex and incompletely understood. Recent ‘omics’ technologies help to transform our understanding of cellular diversity and function in health and disease. In this review we point out the current state of knowledge on phenotypical and functional changes of monocytes and macrophages during cirrhosis evolution in different compartments and their role in disease progression. We also discuss the value of potential prognostic markers for cirrhosis-associated immuneparesis, and future immunotherapeutic strategies that may reduce the need for transplantation and death.

## 1 Introduction

Cirrhosis of the liver is a global health burden with increasing prevalence and a high morbidity and mortality causing more than one million deaths per year worldwide (Mokdad et al., 2014; Tsochatzis et al., 2014). Cirrhosis represents a multisystemic disease, that not only involves the liver, but affects multiple organs, diverse immune cells and soluble factors (Ber nsmeier et al., 2020). Chronic liver injury is most frequently caused by alcohol use, nutritive fat accumulation and viral infections and associated with the activation and alteration of liver resident and infiltrating immune cells. It leads to replacement of healthy parenchyma by fibrotic tissue. This results in progressive fibrosis, disrupted liver architecture, aberrant regeneration, inflammation and vascular changes (Marcellin and Kutala, 2018). These processes moreover lead to portal hypertension (PH), an increase in the pressure within the portal vein, which carries blood from the digestive organs to the liver, and porto-systemic shunting, and results in pathological bacterial translocation (BT) (D'Amico et al., 2018; Mandorfer et al., 2020). The clinical course of cirrhosis when patients progress from compensated to decompensated cirrhosis relates to adverse prognosis. In compensated mostly asymptomatic cirrhosis mortality remains low, but dramatically increases in decompensated cirrhosis when ascites, variceal bleeding, bacterial infections, and organ failure develop (Arvaniti et al., 2010; D'Amico et al., 2006). Therapeutic options are limited to the treatment of the underlying cause of cirrhosis and in end stage liver disease transplantation remains the last option (Asrani et al., 2019). Donor organs are limited and don’t meet the demand, therefore the development of new effective therapies for cirrhosis patients preventing disease progression could improve clinical outcomes. Immune dysfunction seen in cirrhosis starts with the onset of chronic inflammation originating from damage associated molecular patters (DAMPs) released from the injured liver, and worsens with systemic pathogen associated molecular pattern (PAMP) exposure due to pathological BT. The underlying mechanisms remain incompletely understood, severe immune exhaustion develops following factors precipitating acute decompensation (AD) or acute-on-chronic liver failures (ACLF), mainly infection (Moreau et al., 2013). The liver is a tolerogenic organ with antimicrobial surveillance function between the portal and systemic circulation, governed by innate, and amplified by adaptive immunity. A better understanding of monocyte and macrophages dysfunction at a molecular level is required in order to evaluate potential immunotherapeutic approaches for the future treatment of cirrhosis.

In this article we aim to point out the importance of monocytes and macrophages in the context of cirrhosis. We review the current state of knowledge which mechanisms contribute to the progressing immune dysfunction from early asymptomatic stages to end-stage liver disease by dissecting the phenotypical and functional changes of monocytes and macrophages from patients with cirrhosis in different compartments. In the following we 1) summarise the current concept of the pathophysiology of cirrhosis as systemic inflammatory condition, 2) dissect the differentiation of monocytes and macrophages in context of cirrhosis and 3) discuss potential biomarkers and potential future immunotherapeutic targets modulating monocyte and macrophage function.

## 2 Cirrhosis

The main underlying aetiologies for cirrhosis in developed countries are alcohol use causing alcohol-related liver disease (ARLD), non-alcoholic fatty liver disease (NAFLD)—recently also referred to as metabolic-associated fatty liver disease (MAFLD) (Eslam et al., 2020)—and viral infections such as chronic hepatitis B and C (HBV/HCV) (Asrani et al., 2019). In Europe the aetiologies causing cirrhosis are changing due to the therapeutic cure of HCV and the control of chronic HBV, the increasingly widespread unhealthy use of alcohol, the growing epidemic of obesity, and undiagnosed or untreated liver disease (Karlsen et al., 2022). It is believed that NAFLD will become the most common aetiology for liver transplantation in end stage cirrhotic patients (Haldar et al., 2019; Younossi et al., 2019). Cirrhosis has been defined by severe parenchymal loss due to necrosis, inflammation and fibrogenesis as a consequence of diverse underlying aetiologies. It is histopathologically characterised by diffuse nodular regeneration fibrous septa, parenchymal loss and vascular remodelling (Tsochatzis et al., 2014). Pathophysiologically, it involves the accumulation of fibrous tissue due to the activation of heaptic stellate cells (HSCs), as well as changes in the vascular architecture of the liver, leading to increased resistance to portal blood flow and progressive PH (Wanless, 2020). Over the previous 2 decades, it has been shown that under certain circumstances fibrosis and even cirrhosis may be reversible. These conditions must involve an effective and sustainable treatment of the underlying cause of liver injury (Wanless et al., 2000; Lee et al., 2015a; Hytiroglou and Theise, 2018) e.g., successful treatment of chronic viral hepatitis (Chang et al., 2010; Marcellin et al., 2013).

### 2.1 Disease stages of cirrhosis

**FIGURE 1:**
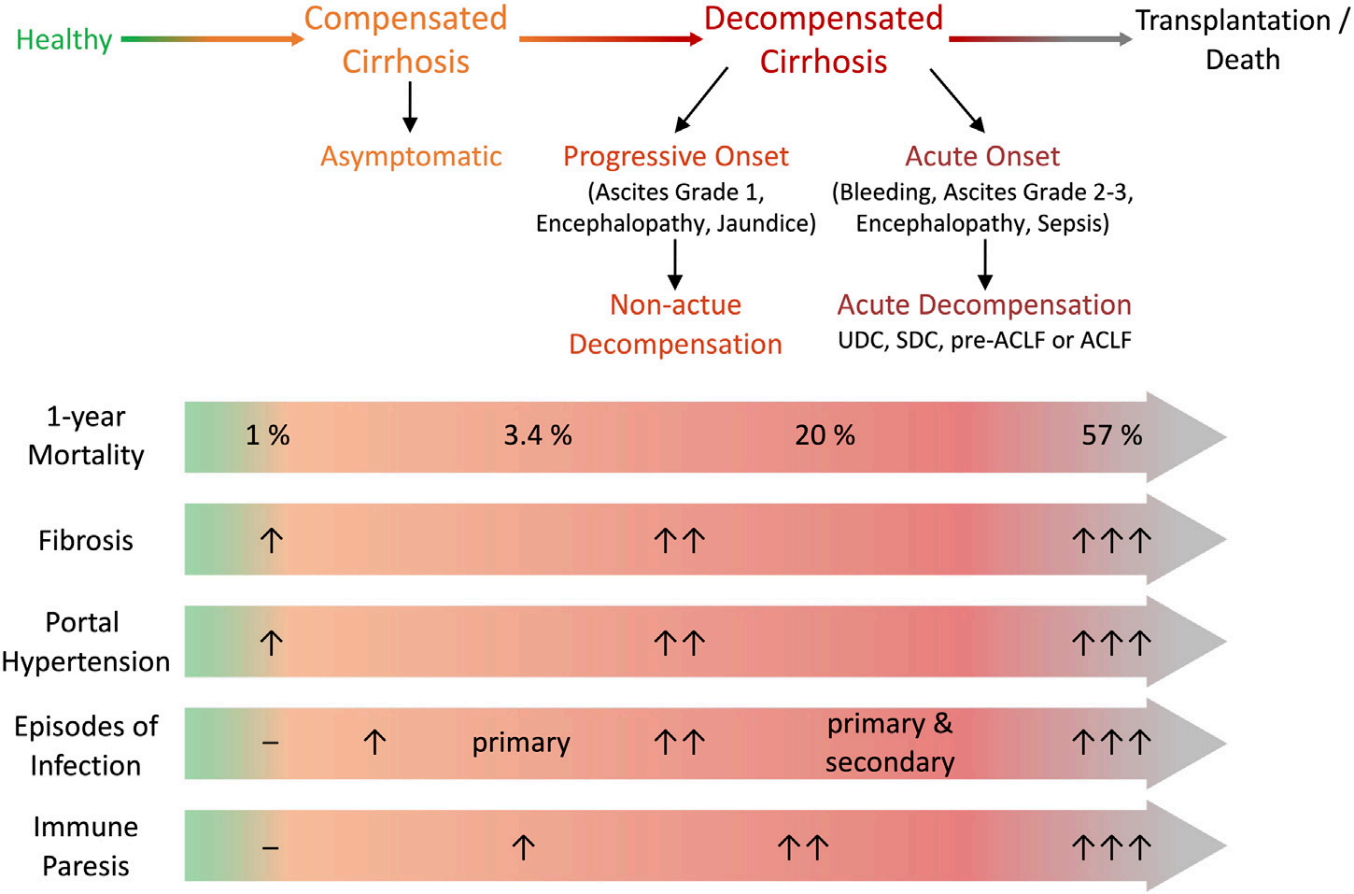
Definitions of decompensation and clinical stage of cirrhosis. Cirrhosis can be categorized in an asymptomatic compensated stage and decompensated stages with the onset of various complications. Decompensation can be characterised by progressive non-acute onset (non-acute decompensation, NAD) or acute onset (acute decompensation, AD). Progressive onset is defined as slow formation of ascites, low grade encephalopathy or progressive jaundice and doesn’t necessarily require hospitalization. AD is defined as rapid ascites formation within less than 2 weeks, acute hepatic encephalopathy, gastrointestinal bleeding or bacterial infection and usually requires hospitalization. AD presents as SDC with stable decrease of systemic inflammation and no further AD for at least 1 year, UDC characterised by persistent albeit unstable inflammatory status resulting in further AD events within 1 year, pre-ACLF with ACLF occurring within 3 months and ACLF defined by development of organ failure. During progression of cirrhosis mortality, fibrosis, portal hypertension and its associated clinical manifestations, the risk for episodes of infection increases and relates to the development of immune paresis. Adapted from (D’Amico *et al.,* Journal of Hepatology 2022 and Bernsmeier *et al.,* Journal of Hepatology 2020). ACLF, acute-on-chronic liver failure; SDC, stable decompensated cirrhosis; UDC, unstable decompensated cirrhosis.

Liver cirrhosis can roughly be categorized into a typically asymptomatic compensated stage and a decompensated stage, marked by the occurrence of complications and reduced survival (D'Amico et al., 2006; Moreau et al., 2013; Planas et al., 2006; Arroyo et al., 2020). However, this classification does not discriminate between the prognostic subgroups that characterise the course of decompensation, which depends on the type and number of decompensating events (D’Amico et al., 2022) (Figure 1). Decompensation of cirrhosis was recently defined by the presence or history of any complication of ascites, bleeding, hepatic encephalopathy (HE) or jaundice (D’Amico et al., 2022), and may be characterised by an acute onset (AD) (Moreau et al., 2013) or by a progressive, non-acute onset (NAD) (D’Amico et al., 2022) (Figure 1). NAD involves slow ascites formation, mild HE, or progressive jaundice in non-cholestatic cirrhosis free of other complications and is mostly represented by first decompensation events that do not require hospitalisation (D’Amico et al., 2022) (Figure 1). In contrast, AD is defined as any first or recurrent moderate or severe ascites within less than 2 weeks, first or recurrent acute HE in patients with previous normal consciousness, acute gastrointestinal bleeding, and any type of acute bacterial infection (D’Amico et al., 2022; Trebicka et al., 2021; Arroyo et al., 2021). AD may manifest itself as unstable decompensated cirrhosis (UDC), stable decompensated cirrhosis (SDC), pre-ACLF or ACLF (D’Amico et al., 2022). UDC involves a 3-months mortality of 21–35,6%, while SDC occurs with a 3-months mortality of 0–9.5% (D’Amico et al., 2022). In a pre-ACLF state ACLF develops within a 3-months period and shows a 3-months mortality of 53,7–67,4% (D’Amico et al., 2022) (Figure 1). ACLF, the most severe stage of cirrhosis with a 28-days mortality of up to 77% (D’Amico et al., 2022), is defined by the development of organ failures (liver, kidney, brain, circulation, coagulation and lung) (Moreau et al., 2013; Gustot et al., 2015; Arroyo et al., 2020; Bajaj et al., 2021; Trebicka et al., 2021). Overall, the classification of cirrhosis stages has recently developed and remains debated. Consequently, not all publications refer to the same classification. The exact assignment to the stages of cirrhosis studied is highly relevant regarding the interpretation of experimental research data in the field.

**FIGURE 2:**
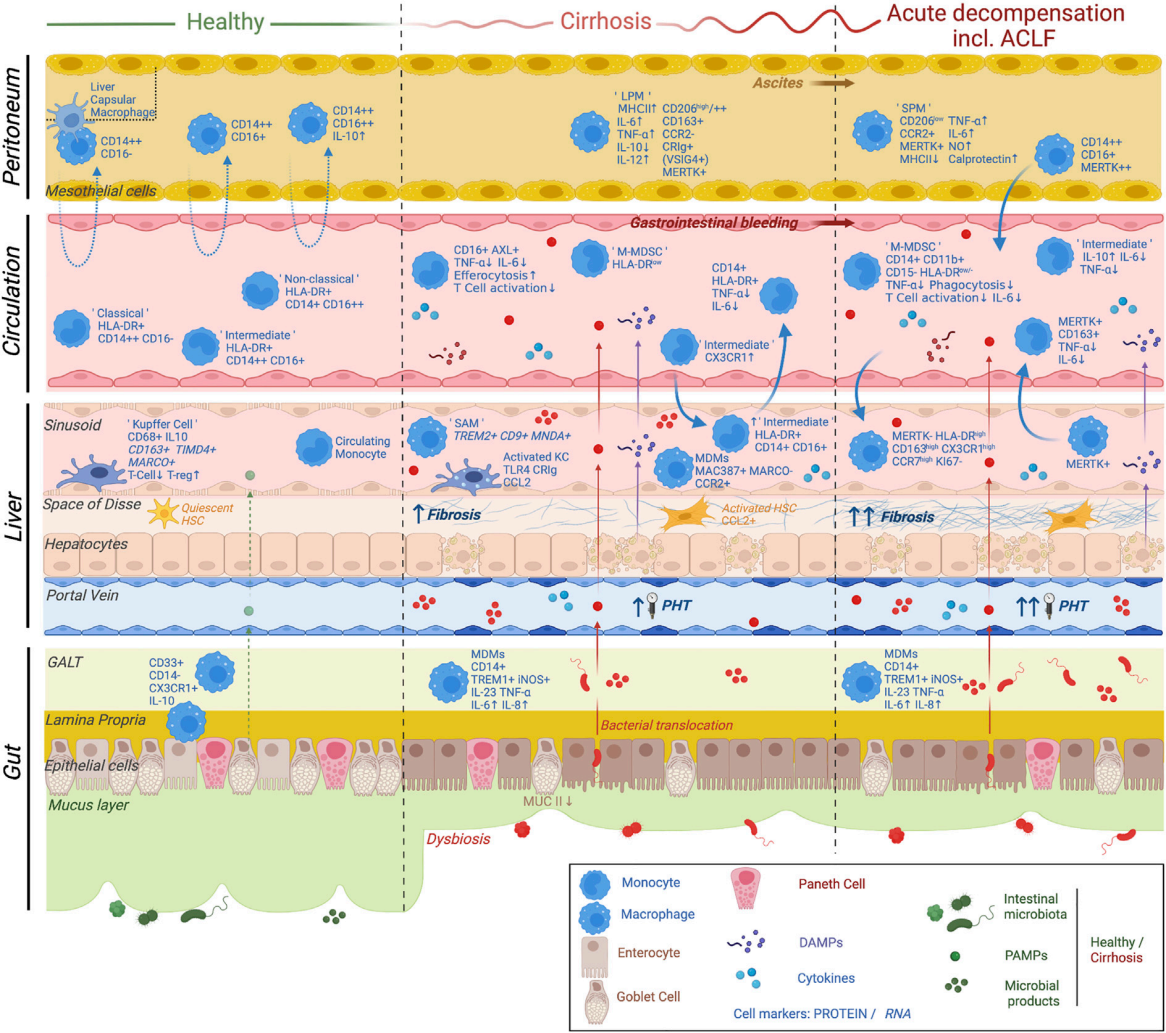
Current knowledge on monocyte and macrophage plasticity in relation to the multi-compartmental pathophysiological mechanisms underlying cirrhosis progression and decompensation. The main pathophysiological mechanisms underlying cirrhosis progression and acute decompensation are shown. These include liver fibrosis, hepatic endothelial dysfunction, parenchymal damage, portal hypertension, dysbiosis, bacterial translocation as well as activation of both local and systemic inflammatory responses. A variety of cellular and non-cellular players involved in these mechanisms are depicted (see symbol legend). In summary, homeostatic monocyte and macrophage populations display a distinct phenotype and function in a compartment-specific manner. Changes in monocyte/macrophage differentiation and migration patterns with progression of cirrhosis and acute decompensation are due to multiple factors involving chronic inflammation, exposure to DAMPs (released by injured hepatocytes) as well as exposure to PAMPs/microbial products due to dysbiosis and pathological bacterial translocation. Distinct cell subsets are shown in gut, liver, circulation and peritoneum, underlining the multi-compartmental nature of the disease. Adapted from Bernsmeier *et al.*, Journal of hepatology 2020. ACLF, acute-on-chronic liver failure; LPM, large peritoneal macrophage; SPM, small peritoneal macrophage; M-MDSC, monocytic myeloid-derived suppressor cell; SAM, scar-associated macrophage; MDM, monocyte-derived macrophage; HSC, hepatic stellate cell; PHT, portal hypertension; GALT, gut-associated lymphoid tissue; DAMP; damage-associated molecular patterns; PAMP, pathogen-associated molecular pattern. Created with BioRender.com.

### 2.2 Pathophysiology of cirrhosis—the systemic inflammation hypothesis

The most important factors in the pathophysiology of immuneparesis in cirrhosis are DAMPs, PAMPs and the systemic inflammatory response (SIRS): Chronic liver injury results in release and exposure to DAMPs, with cirrhosis progression pathologic BT develops and can lead to infections subsequently resulting in SIRS.

#### 2.2.1 Chronic liver injury and DAMPs

Importantly, the pathophysiology of cirrhosis is not restricted to the liver, rather it represents a systemic condition involving various compartments, immune cells and soluble factors (Albillos et al., 2014; Ber nsmeier et al., 2020). Upon chronic liver injury fibrosis occurs and its formation involves activated resident or recruited macrophages, hepatocytes and HSCs. HSCs are key players in fibrosis development as they remain quiescent under homeostatic conditions but become activated following hepatic injury and transform towards myofibroblasts which secrete extracellular matrix proteins (Tsuchida and Friedman, 2017; Kisseleva and Brenner, 2021). Immune dysfunction in cirrhosis initiates with the onset of chronic hepatic inflammation and worsens along with the progression of cirrhosis and the development of PH (Figure 1). It involves components of the innate immune system, which constitutes the first line of defence against pathogens as it plays a key role in maintaining homeostasis by phagocytosis and killing of bacteria, antigen presentation, inflammatory cytokine production, activation of adaptive immune cells and providing physical and chemical barriers. In cirrhosis chronic inflammation, parenchymal damage and scarring of the liver activate local tissue inflammation and contribute to a systemic inflammatory response mediated by DAMPs, cytokines and migration of immunoregulatory innate immune cells (Ber nsmeier et al., 2020). The liver sinusoidal endothelium is highly affected in cirrhosis due to its capillarization and angioarchitectural change (DeLeve, 2015; Su et al., 2021).

#### 2.2.2 Bacterial translocation and PAMPs

The gut-liver axis is an operative unit protecting the body against harmful substances and microorganisms, maintaining homeostasis of the immune system. Liver cirrhosis profoundly alters this complex system and the intestine becomes more permeable allowing the translocation of bacteria and bacterial products (PAMPs) into the portal circulation (Ponziani et al., 2018). In the gut, dysbiosis, i.e. the reduction of beneficial taxa and increase in pathogenic bacteria, and reduction of the mucus layer is observed (Bonnel et al., 2011; Albillos et al., 2014; Bajaj et al., 2014; Wiest et al., 2017; Fernández et al., 2018; Fernández et al., 2019; Piano et al., 2019; Albillos et al., 2020; Bajaj et al., 2021; Van der Merwe et al., 2021). Gut endothelial and vascular barrier dysfunction results in BT, endotoxemia as well as mucosal immune cell alteration (Zapater et al., 2008; Ber nsmeier et al., 2020; Haderer et al., 2022). Pathological BT, present in advanced cirrhosis, is defined by the increased rate of translocation of bacteria or bacterial products from the gut to mesenteric lymph nodes, the portal and systemic circulation as well as other tissues. Gut derived PAMPs further drive hepatic inflammation and immune cell differentiation in the gut, liver, circulation and other organs (Zapater et al., 2008; Ber nsmeier et al., 2020; Stengel et al., 2020; Haderer et al., 2022).

#### 2.2.3 Onset of infections and acute decompensation

Patients with cirrhosis are at high risk of bacterial infections, which are associated with the development of AD, hospitalisation, sepsis and the need for intensive care and organ support involving a substantial mortality (Arvaniti et al., 2010; Moreau et al., 2013; Jalan et al., 2014; Van der Merwe et al., 2021; Triantos et al., 2022) (Figure 1). The most common infections include spontaneous bacterial peritonitis (SBP), urinary tract infection, pneumonia, spontaneous bacteraemia, skin, and soft tissue infections (Arvaniti et al., 2010; Bajaj et al., 2012; Fernández et al., 2018; Piano et al., 2019; Arroyo et al., 2021; Trebicka et al., 2021). Bacterial infections in hospitalised patients are much more frequent in patients with cirrhosis (32–34%) compared to those without (5–7%). Remarkably, around 45% of hospitalised cirrhosis patients with gastrointestinal bleeding develop infections (Borzio et al., 2001; Tandon and Garcia-Tsao, 2008; Arvaniti et al., 2010). Of note, it has been observed that a dual infection (bacterial and fungal) increases the mortality of cirrhosis patients compared to patients with a single infection (Bajaj et al., 2018; Bajaj et al., 2021). As in the general population, infections caused by multi-drug-resistant organisms are progressively increasing in cirrhotic patients and represent up to 30–35% of all infections (Fernández et al., 2012; Fernández et al., 2019; Piano et al., 2019; Onorato et al., 2022).

Pathological BT in the context of immuneparesis can lead to SBP (Assimakopoulos et al., 2012; Wiest et al., 2014). SBP is diagnosed in patients with an elevated neutrophil count in the ascitic fluid (Runyon and Aasld, 2013), but no differences in mortality were observed independently of whether the bacterial culture of ascites was positive or negative (Runyon and Aasld, 2013). Moreover, BT and gut barrier impairment are directly related to the risk of variceal bleeding (Triantos et al., 2022). The plasticity of this process is accompanied by SIRS activation in ACLF and implicates changes in neural regulation, circulating inflammatory mediators and hormones (Bosmann and Ward, 2013; Bernsmeier et al., 2015a). Sepsis and SIRS are associated with hyperinflammation, impaired innate immune function, immunosuppression and complement activation, collectively leading to septic shock and organ failure (Bosmann and Ward, 2013).

Infection susceptibility in cirrhosis has been attributed to a state of immuneparesis, defined by inadequate immune responses to microbial challenge (Bonnel et al., 2011; Bajaj et al., 2012; Albillos et al., 2014). The pathophysiology of immuneparesis is very complex and incompletely understood, involving diverse defects in immune cell function, including monocytes and macrophages in multiple compartments (Bonnel et al., 2011). This suggests that the disease specific differentiation and function of monocytes and macrophages actively contribute to inflammation and infection susceptibility in cirrhosis patients. The following sections focus on the plasticity of mononuclear phagocytes in the context of cirrhosis.

## 3 Immunepathophysiology of mononuclear phagocytes in cirrhosis

Mononuclear phagocytes (monocytes and macrophages) play a crucial role in initiating immune responses. Their functions include phagocytosis, killing of bacteria, antigen presentation, cytokine production, immune cell recruitment to sites of infection, and inflammation and activation of immune effector cells. Phagocytes regulate the antimicrobial defence, inflammation in tissue injury, fibrogenesis and tumourigenesis (Yona et al., 2013; Ziegler-Heitbrock and Hofer, 2013). Related to these functions, monocytes and macrophages are essential in the pathophysiology of immune dysfunction in patients with cirrhosis of the liver as a systemic inflammatory condition (Ber nsmeier et al., 2020; Singanayagam and Triantafyllou, 2021) (Figure 2).

### 3.1 Circulating monocytes

Principally, monocytes can be divided into classical (CD14^++^CD16^−^), intermediate (CD14^++^CD16^+^) and non-classical (CD14^+^CD16^++^) subsets (Passlick et al., 1989; Jakubzick et al., 2017; Patel et al., 2017; Guilliams et al., 2018; Kapellos et al., 2019) and further identified by their human leukocyte antigen DR (HLA-DR) expression (Guilliams et al., 2018). The intermediate and non-classical monocyte subsets emerge sequentially from the pool of classical monocytes (Patel et al., 2017). It has been found in mice and confirmed in human that CD14^+^CD16^++^ non-classical monocytes fulfil a continuous monitoring of the vasculature by a crawling mechanism on endothelial cells (Cros et al., 2010). This behaviour permits the efficient scavenging of luminal microparticles under homeostatic conditions. While this established classification was based on protein assays which involved mainly flow cytometry-based techniques, recent unbiased single-cell RNA sequencing (scRNA-Seq) studies have suggested broader heterogeneity within the intermediate population of monocytes, through the identification of two distinct intermediate monocyte subsets, one expressing classical monocyte and cytotoxic genes, while the other one with so far unknown functions (Villani et al., 2017).

#### 3.1.1 Monocyte development

The development of classical monocytes was firstly discovered with the use of mouse models and some of these findings could subsequently be transferred to humans. In mice, haematopoietic stem cells in the bone marrow give rise to a monocyte/dendritic cell progenitor (MDP) (Guilliams et al., 2018). This MDP population was proposed to give rise to monocytes and classical dendtritic cells (DCs) in both mice (Olsson et al., 2016; Guilliams et al., 2018) and humans (Lee et al., 2015b; Guilliams et al., 2018), by following a binary trajectory towards either dedicated common DC precursors (CDP) or into unipotent common monocyte progenitors (cMoP) (Lee et al., 2015b; Guilliams et al., 2018). Accordingly, the cMoP population has also been identified in human bone marrow (BM) (Kawamura et al., 2017). An important transcription factor maintaining commitment towards the monocyte lineage in humans is interferon regulatory factor 8 (IRF8) (Hambleton et al., 2011). An autosomal recessive IRF8 deficiency in humans leads to reduced numbers of circulating monocytes (Hambleton et al., 2011). Interestingly, investigations into cMoP biology have uncovered that this population possesses a high proliferative capacity and is characterized by CD14 expression in humans (Chong et al., 2016). Further differentiation of cMoP into mature monocytes involves a transient pre-monocyte stage, discriminated by the expression of CXC-motiv chemokinereceptor 4 (CXCR4) in mice and humans (Chong et al., 2016). In mice the downregulation of Cxcr4 expression is accompanied by the up-regulation of C-C chemokine receptor 2 (Ccr2), indicating that Ccr2 regulates the BM exit of murine monocytes (Serbina and Pamer, 2006; Chong et al., 2016). While it remains unclear whether CCR2 also plays a role in BM exit of human monocytes to the circulation (Guilliams et al., 2018), it was shown that CXCR4 participates in the homing of classical and non-classical monocytes to central (BM) and peripheral (spleen) monocyte reservoirs (Chong et al., 2016). In mice, following release of classical monocytes into the circulation from the BM under healthy homeostasis, these cells remain in the circulation and may subsequently either traffic to repopulate a proportion of tissue-resident macrophages (Tamoutounour et al., 2012; Bain et al., 2013; Jakubzick et al., 2013; Bain et al., 2014; Epelman et al., 2014; Calderon et al., 2015; Guilliams et al., 2018) or alternatively convert into non-classical monocytes (Yona et al., 2013; Patel et al., 2017). In an analogous manner, it was observed in humans that the vast majority of classical monocytes are recruited to peripheral tissues where they potentially differentiate into monocyte-derived cells or enter reservoirs of undifferentiated monocytes (Tak et al., 2017a; Patel et al., 2017). The transition of classical monocytes to non-classical monocytes has been observed in different species including mouse (Sunderkötter et al., 2004; Tacke et al., 2006; Varol et al., 2007; Yona et al., 2013), rat (Yrlid et al., 2006), macaque (Sugimoto et al., 2015) as well as in humans (Patel et al., 2017) and therefore represents an evolutionarily conserved program (Guilliams et al., 2018).

#### 3.1.2 Monocyte differentiation in the context of inflammation and cirrhosis

Proportions of circulating monocytes vary depending on monopoiesis, tissue infiltration and their release from central (BM) or peripheral reservoirs. In humans, endotoxin induces a rapid yet transient monocytopenia during the first 2 h after lipopolysaccharide (LPS) injection followed by the sequential reappearance of CD14^++^CD16^−^ classical monocytes, followed by CD14^+^CD16^+^ intermediate cells and finally CD14^+^ CD16^++^ non-classical monocytes (Thaler et al., 2016; Tak et al., 2017b; Patel et al., 2017). The number of non-classical monocytes is thus strongly linked to the physiological status of the organism and therefore represents a potential diagnostic tool (Selimoglu-Buet et al., 2015). Not only do cytokines directly provoke functional changes in monocytes, but they also influence the cellular outcome of haematopoietic stem cells, a phenomenon called “trained immunity” (Quintin et al., 2012; Netea et al., 2016). A number of cytokines have been proposed to play a key role in trained immunity, including interferon-ɣ (INF-ɣ) and interleukine-1β (IL-1β), suggesting that activation of haematopoietic stem cells by cytokines produced by immune or non-immune cells in the BM is crucial for these long-lasting training effects (Boettcher and Manz, 2017). Inflammation-induced emergency haematopoiesis can result in trained immunity characterized by long-term epigenetic effects on haematopoietic stem cells. The epigenetic changes associated with monocyte training involve histone modification of genes encoding pro-inflammatory cytokines such as IL-6, tumor necrosis factor-α (TNF-α) but also genes of the mammalian target of rapamycin (mTOR) pathway (Quintin et al., 2012; Netea et al., 2016). Under pathological conditions, monocytes gain distinct non-redundant functions that often cannot be fulfilled by resident macrophages and DCs. These non-exclusive and sometimes overlapping effector functions comprise pro-inflammatory activities, antigen-presentation, tissue remodelling, or anti-inflammatory abilities (Patel et al., 2017; Guilliams et al., 2018). Furthermore, in mouse models effector monocytes actively participate in fibrosis development (Satoh et al., 2017) and can also differentiate into pathogenic foam cells (Guilliams et al., 2018). This highlights that monocytes are not simply precursors that get recruited to temporarily increase the number of tissue resident macrophages but in fact give rise to functionally distinct monocyte-derived cells (Guilliams et al., 2018).

In patients with cirrhosis of the liver the number of circulating monocytes increases gradually with the progression of disease (Hassner et al., 1979; Zimmermann et al., 2010; Korf et al., 2019a; Brenig et al., 2020). An expansion of the intermediate monocyte population was observed in the circulation and the liver in the context of cirrhosis progression, as a consequence of enhanced recruitment from blood and local differentiation from classical monocytes (Zimmermann et al., 2010; Liaskou et al., 2013; Korf et al., 2019a). This indicates the pathological condition present in patients with cirrhosis is reflected by monocyte differentiation. Intermediate CD14^++^CD16^+^ monocytes accumulated in chronically inflamed and cirrhotic human liver as a consequence of enhanced recruitment from the blood (Zimmermann et al., 2010; Liaskou et al., 2013; Zimmermann et al., 2016). These monocytes displayed a high level of phagocytosis, antigen presentation, T cell proliferation and secretion of cytokines (TNF-a, IL-6, IL-8, and IL-1b) as well as profibrogenic cytokines (IL-13) and growth factors (granulocyte-colony stimulating factor G-CSF, granulocyte-macrophage-colony stimulating factor GM-CSF) (Liaskou et al., 2013).

#### 3.1.3 Expansion of M-MDSC in cirrhosis

One of the first discoveries of monocyte dysfunction in patients with cirrhosis was their impaired phagocytosis capacity (Sanchez-Tapias et al., 1977; Sherlock, 1977; Hassner et al., 1979; Holdstock et al., 1982). In line with this finding, a reduced expression of major histocompatibility complex class II (MHC II) molecules was observed on monocytes from patients with ACLF and was associated with adverse prognosis (Hassner et al., 1979; Wasmuth et al., 2005). Confirming these findings, it was shown that MHC II molecule expression gradually decreased with cirrhosis progression (Lin et al., 2007; Xing et al., 2007; Bernsmeier et al., 2018; Brenig et al., 2020). HLA-DR, a MHC II family member, can be considered as a monocyte activation marker, due to the increased expression upon immune stimulation and its function in inducing T cell responses (Gordon and Taylor, 2005). On the contrary, the downregulation of HLA-DR appears in acquired immunosuppressive monocyte differentiation (Gordon and Taylor, 2005).

The reduced HLA-DR expression on monocytes in cirrhosis was associated with the expansion of mononuclear myeloid-derived suppressor cells (M-MDSC, CD11b^+^CD14^+^HLA-DR^lo/−^CD15^−^) (Bronte et al., 2016) in the circulation of patients with cirrhosis (Bernsmeier et al., 2018). The term MDSCs was first introduced in the year 2007 in a cancer-related context (Gabrilovich et al., 2007). MDSCs develop from myeloid cell expansion and conditioning in the BM and spleen with subsequent conversion of monocytes into pathologically activated MDSCs (Condamine et al., 2015; Wang et al., 2019; Ostrand-Rosenberg et al., 2020). Human M-MDSCs are distinct from monocytes by their low expression of MHC II (Bronte et al., 2016). The main characteristic of MDSCs is the inhibition of immune responses, including those mediated by T cells, B cells and NK cells (Gabrilovich, 2017). The suppression of immune responses is mediated by upregulation of signal transducer and activator of transcription 3 (STAT3) expression, induction of endoplasmic reticulum (ER) stress, expression of arginase 1 (Arg1) and expression of S100A8/A9 (Veglia et al., 2021). To fulfil their immunosuppressive function M-MDSCs use nitric oxide (NO), immunosuppressive cytokines such as interleukin-10 (IL-10) and transforming growth factor β (TGF-β) and the expression of immune regulatory molecules like programmed death-ligand 1 (PD-L1) (Gabrilovich, 2017). Recently, trajectory analysis showed that M-MDSCs are also molecularly distinct from M1-like and M2-like macrophages (Song et al., 2019), indicating that M-MDSCs represent a subset distinct from monocytes and macrophages.

In the context of cirrhosis, endotoxemia (the presence of bacteria-derived endotoxins in the blood stream) is observed in advanced stages of disease and is associated with the loss of HLA-DR (Zapater et al., 2008; Bernsmeier et al., 2018). Moreover, loss of HLA-DR expression affects the antigen presentation capacity of monocytes and their ability to induce activation of adaptive immune responses and is associated with adverse clinical outcomes in patients with cirrhosis (Bernsmeier et al., 2018; Brenig et al., 2020). M-MDSCs in AD/ACLF patients displayed immunosuppressive properties in regard to their pro-inflammatory cytokine production (TNF-α, IL-6) (Wasmuth et al., 2005; Lin et al., 2007; Berres et al., 2009; Berry et al., 2011; Bernsmeier et al., 2018; Korf et al., 2019a; Brenig et al., 2020) as well as their reduced T cell activation and decreased phagocytosis capacity, contributing to the dampened innate immune responses of circulating monocytes (Bernsmeier et al., 2018). Sequentially increasing numbers of M-MDSC with disease progression from compensated cirrhosis to AD/ACLF (Bernsmeier et al., 2018) underlie the low TNF-α/IL-6 responses in AD/ACLF patients. Furthermore CD8^+^ T cells from cirrhotic patients displayed high HLA-DR, T cell immunoglobulin and mucin-domain containing-3 (TIM-3), cytotoxic T-lymphocyte-associated protein 4 (CTLA-4) and programmed cell death protein 1 (PD-1) expression associated with concomitant infections and disease severity (Lebossé et al., 2019).

*In vitro* exposure of monocytes from healthy controls to plasma isolated from AD/ACLF patients reduced monocytic HLA-DR expression as well as suppressed TNF-α/IL-6 responses. (Bernsmeier et al., 2018; O'Brien et al., 2014; Bernsmeier et al., 2015b). In a similar fashion healthy human monocytes exposed to activated primary human HSCs, converted mature CD14^+^ monocytes into M-MDSCs in a contact-dependent manner (Höchst et al., 2013; Resheq et al., 2015). Recently it has been shown that MDMCs from patients with non-cirrhotic chronic HBV infection had profound suppressive ability, expressing Arg1/iNOS/PD-L1/CTLA-4/CD40 at significantly greater levels relative to healthy controls (Pal et al., 2022).

#### 3.1.4 TAM receptor expression on monocytes in cirrhosis

Lately, TAM receptors (TYRO3, AXL, and MERTK), which are tyrosine kinase receptors expressed on monocytes and macrophages and essential for the regulation of immune homeostasis, have been shown to play a central role in cirrhosis and contribute to the systemic immune paresis in patients with cirrhosis and liver failure (Roth lin et al., 2007; Bernsmeier et al., 2015b; Triantafyllou et al., 2018; Brenig et al., 2020; Flint et al., 2022). They maintain immune homeostasis by regulating innate immune responses via suppression of inflammatory toll-like receptor (TLR) signalling cascades and promotion of tissue resolution thorough the clearance of apoptotic cells (efferocytosis) (Flint et al., 2022). Immuneregulatory AXL-expressing monocytes expanded along with the progression of cirrhosis, representing <5% in early stages and up to 40% of monocytes in later stages, but were scarce following an event triggering AD (<5%) (Brenig et al., 2020). In compensated cirrhosis and NAD, the expansion of AXL-expressing circulating monocytes correlated with the occurrence of infectious episodes, onset of subsequent AD within 4 months and 1-year mortality (Brenig et al., 2020). On a functional level, AXL-expressing monocytes exhibited a reduced T cell activation potential and dampened TNF-α and IL-6 responses upon TLR4 stimulation through LPS treatment. However, AXL^+^ monocytes showed preserved capacity to phagocytose pathogens and enhanced capacity to phagocyte apoptotic cells, i.e. efferocytosis, when compared to AXL^−^ monocytes (Brenig et al., 2020). Another TAM receptor family member, MERTK was found to be up-regulated on circulating monocytes in the stage of AD/ACLF in cirrhosis patients (Bernsmeier et al., 2015b; Brenig et al., 2020). MERTK-expressing monocytes represented 14.4% of all circulating monocytes in patients with AD and even 35.7% in ACLF, respectively, and showed dampened innate immune responses to LPS in close association with clinical disease severity scores and the need for transplantation or death (Bernsmeier et al., 2015b).

#### 3.1.5 Functional consequences of monocyte plasticity following acute decompensation

Monocytes from patients with ACLF showed polarisation towards an immunotolerant state by reduced HLA-DR expression, reduced TNF-α/IL-6 production but elevated IL-10 production (Wasmuth et al., 2005; Lin et al., 2007; Berres et al., 2009; Berry et al., 2011; Bernsmeier et al., 2018; Korf et al., 2019a; Brenig et al., 2020), and impaired phagocytic capacity. As indicated, the phagocytic capacity of monocytes was maintained during disease progression, but diminished following insults leading to AD/ACLF stages (Gadd et al., 2016; Bernsmeier et al., 2018).

Under pathological conditions the number, phenotype and function of circulating monocytes changes, indicating that monocytes are not only macrophage precursors, but in fact form a separate cell population giving rise to functionally distinct cells. Immunosuppressive M-MDSCs increase in late stages of cirrhosis either developing earlier in the BM and spleen or originating from mature circulating monocytes in the disease-specific milieu. The appearance of distinct monocyte subsets at early and late stages of disease indicates that monocyte subsets are susceptible to phenotypic and functional changes not only on a mature circulating monocyte level, but already on a developmental level. Thus, monocyte differentiation is continually adapting to the inflammatory milieu in response to PAMPs, DAMPs and SIRS. In NAD, AD, and ACLF stages of disease, immunoregulatory monocytic populations expanding and replace substantial proportions of functionally regular counterparts. In order to prevent infection, decompensation and death from cirrhosis, it is desirable to further explore therapeutic options aimed at restoring systemic innate immune homeostasis by reconstituting monocyte function, while also assessing the risk of therapy-related side effects (see Section 5).

### 3.2 Macrophages in the liver

Monocytes are considered to be precursors of macrophages polarizing and generating subpopulations traditionally called activated, pro-inflammatory macrophages “M1” and alternatively activated, anti-inflammatory macrophages “M2” (Nathan et al., 1983; Mosmann et al., 1986; Murray et al., 2014; Chávez-Galán et al., 2015). This categorization of macrophages cannot be applied to hepatic macrophages since they simultaneously express M1 and M2 markers (Gao et al., 2019) and recent evidence using mouse models suggested that liver resident Kupffer cells (KCs) are self-renewing and originate from the yolk sack (Li et al., 2017). These cells exhibit great plasticity which is dependent on their ontogeny, local and systemic microenvironmental mediators and epigenetic programming (Guillot and Tacke, 2019; Wen et al., 2021). To date, markers that distinctly identify KCs in humans and allow to distinguish these cells from monocyte-derived macrophages (MDMs) have been reviewed. Undoubtedly, new technical opportunities provided by ‘omics’ technologies (genomics, transcriptomics, proteomics, and metabolomics) will allow for the re-evaluation and further exploration of distinct KC and MDM markers (Guilliams et al., 2022). The liver drains blood from abdominal organs through the portal vein and receives systemic blood via the hepatic artery (Jenne and Kubes, 2013). It is a highly organised tolerogenic organ with a large sinusoidal network. These so-called sinusoids are lined by permeable fenestrated liver sinusoidal endothelial cells (LSECs) enabling the exposure of plasma content such as gut-derived bacterial products to hepatocytes and non-parenchymal cells of the liver (Su et al., 2021) and harbour KCs (Figure 2).

#### 3.2.1 Kupffer cells

In homeostatic conditions immune activation is prevented by KCs, LSEC, and DCs, which together promote immune tolerance. KCs are the most abundant hepatic immune cells maintaining liver homeostasis and immune tolerance, due to their continuous exposure to harmless gut-derived antigens and their ability to rapidly identify and eliminate pathogens (Wen et al., 2021). The key role of liver resident KCs is to phagocytose and scavenge DAMPs, recognize PAMPs and remove circulating bacteria to prevent systemic immune activation by secreting IL-10 (Kubes and Jenne, 2018). Furthermore, KCs are capable of self-renewal and show a specific transcriptional program defined by their unique niche (Zigmond et al., 2014; Beattie et al., 2016; Krenkel and Tacke, 2017). KCs are located within the sinusoidal lumen, in continuous contact with LSECs and can also interact with HSCs and hepatocytes across the space of Disse (Jenne and Kubes, 2013; Bonnardel et al., 2019; Sutti et al., 2019). Liver resident KCs are capable of removing bacterial products and secreting tolerogenic factors, regulating this microenvironment (Sutti et al., 2019). KCs have the ability to inhibit T cell expansion and induce regulatory T cells, that may reprogram liver infiltrating monocytes to regulatory IL-10^+^ DCs that further promote tolerance (Heymann et al., 2015; Heymann and Tacke, 2016). Furthermore, hepatocytes, HSCs and endothelial cells shift infiltrating monocytes towards a more tolerogenic phenotype (Blériot and Ginhoux, 2019; Bonnardel et al., 2019).

The balance between immunity and tolerance is essential to liver function (Kubes and Jenne, 2018) and in cirrhosis the mechanisms to maintain tolerance fail (Wu et al., 2010). Chronic liver injury and inflammation lead to progressive liver fibrosis and ultimately to the development of cirrhosis with formation of fibrous septa, regenerative nodules, sinusoidal resistance, intrahepatic shunting and PH (Koyama and Brenner, 2017; Laleman et al., 2018). The liver, which drains the intestinal venous blood, forms a vascular “firewall” that captures gut commensal bacteria entering the blood stream during intestinal pathology (Balmer et al., 2014). The compartmentalization of intestinal microbes was found to be defective in animal models of liver disease due to failure of the hepatic vascular “firewall” that is required to clear microbes from the mesenteric and systemic vasculature efficiently (Balmer et al., 2014). Mononuclear phagocytic cells in the liver can be categorized into liver resident KCs (Kubes and Jenne, 2018), circulating monocytes trafficking through the sinusoids or migrating monocytes, which may persist as monocytes in the tissue, acquire antigen-presenting capability or mature into macrophages (Strauss et al., 2015; Jakubzick et al., 2017; Weston et al., 2019). During the progression from inflammation with fibrosis to cirrhosis, KCs are continuously activated by DAMPs released by dying hepatocytes. Activated TLR4/complement receptor of the immunoglobulin superfamily (CRIg)-expressing KCs lose their tolerogenic phenotype and secrete pro-inflammatory cytokines, amplifying immune responses and recruiting immune cells to the liver (Kolios, 2006; Weston et al., 2019). Experimental models of acute liver injury in rodents have been used to better understand macrophage function during liver disease (Weston et al., 2019). These models showed that extensive hepatocyte damage releases DAMPs which are sensed by KCs leading to the release of cytokines, chemokines and pro-inflammatory macrophage recruitment (Krenkel and Tacke, 2017). During acetaminophen-induced acute liver injury in humans a phenotypic switch in macrophage phenotype is observed, where MAC387 (S100A9) serves as a marker to identify MDMs in contrast to CD68^+^ resident KCs in the liver (Antoniades et al., 2012). Recently, using scRNA-Seq KCs were phenotypically described as CD68^+^CD163^+^MARCO^+^TIMD4^+^ (MacParland et al., 2018; Aizarani et al., 2019; Ramachandran et al., 2019).

#### 3.2.2 Capsular macrophages

Furthermore, a distinct population of MHC II-expressing cells was observed in the hepatic capsule (PRICKE TT et al., 1988). In mice it has been shown that this population represented a macrophage population (liver capsular macrophages LCMs) that is distinct from KCs (Sierro et al., 2017). LCMs in mice are CX3CR1^hi^TIM4^-^ and replenished in the steady state from blood-borne monocytes (Sierro et al., 2017). Recent spatial transcriptomic experiments in mice have shown that these capsule macrophages can be further characterised by the expression of CD207 and CX3CR1 (Guilliams et al., 2022), but could not be identified in human liver biopsies due to the lack of capsule tissue (Guilliams et al., 2022). In mice, LCMs form a cellular network in the hepatic capsule and potentially sense the blood and the peritoneal cavity due to their extended dendrites (Sierro et al., 2017). It has been suggested that LCMs sense peritoneal bacteria accessing the liver capsule and prevent them from breaching the liver capsule through the recruitment of neutrophils (Sierro et al., 2017).

#### 3.2.3 Monocyte-derived macrophages

In cirrhotic livers the number of intermediate CD14^++^CD16^+^ monocytes increases, these cells accumulate at sites of inflammation and fibrosis and are characterised by enhanced phagocytosis capacity, antigen presentation capacity, activation potential for T cell proliferation and secretion of numerous soluble factors like chemokines, growth factors, pro-inflammatory, and pro-fibrogenic mediators (Zimmermann et al., 2010; Liaskou et al., 2013; Tsuchida and Friedman, 2017). Intermediate monocytes contribute to the perpetuation of hepatic inflammation and fibrogenesis and show an increased CX3CR1-dependent migratory potential towards liver tissue, while also displaying the ability to transmigrate back to the circulation and contribute to SIRS (Aspinall et al., 2010; Zimmermann et al., 2010). In the context of cirrhosis capillarization of LSECs, formation of basement membranes and activation of HSCs contributes to increased migration of immune cells across the sinusoidal endothelial barrier (Bataller and Brenner, 2005; Henderson et al., 2006; Li et al., 2012; Ramachandran et al., 2012; Shetty et al., 2018; Su et al., 2021). Both, activated KCs and HSCs produce CCL2 and CCL5 promoting the recruitment of MDMs to the liver through CCR2/CCL2 or CCR5/CCL5 signalling (Heymann and Tacke, 2016; Ju and Tacke, 2016; Krenkel and Tacke, 2017; Sasaki et al., 2017; Ramachandran et al., 2020). In addition, activated HSCs can also recruit monocytes through the CCR8/CCL1 and CXCR3/CXCL10 axes (Heymann et al., 2012; Tomita et al., 2016; Zhang et al., 2016). However CCR2 expression on KCs is absent under healthy conditions, but increases in parallel to disease evolution, serving as diagnostic marker in the context of NAFLD (Krenkel et al., 2018). CCR2^+^ macrophages are also seen in patients with acetaminophen (APAP)-induced liver failure (Mossanen et al., 2016) and increased serum CCL2 levels are associated with adverse prognosis (Antoniades et al., 2012) serving as a potential biomarker also for ALF, mitigating its specificity. MDMs have been shown to regulate a number of aspects of liver injury, including perpetuation of inflammation (Wu et al., 2016; Krenkel and Tacke, 2017), but also promotion of fibrosis (Karlmark et al., 2009; Krenkel et al., 2018; Weston et al., 2019; Ramachandran et al., 2020). Both KCs and MDMs can adopt context-dependent fibrogenic and fibrolytic roles due to their heterogeneity and plasticity (Weston et al., 2019).

ScRNA-Seq revealed MDMs as CD68^+^MARCO^−^ in comparison to CD68^+^CD163^+^MARCO^+^TIMD4^+^ KCs (MacParland et al., 2018; Aizarani et al., 2019; Ramachandran et al., 2019). A cross-species comparison suggested a conserved transcriptional gene signature among human and mouse KCs (Ramachandran et al., 2019; Ramachandran et al., 2020). Recent publications revealed that expression of TREM-1, an amplifier of inflammation, on macrophages promoted hepatic inflammation and fibrosis in mice and humans (Nguye n-Lefebvre et al., 2018; Perugorria et al., 2019). Moreover, TREM-1 promoted pro-inflammatory cytokine production and mobilization of inflammatory cells to the site of injury (Nguye n-Lefebvre et al., 2018). In human liver samples from patients with severe fibrosis an increase in the number of TREM-1^+^ cells in fibrotic areas was observed, compared to only slight TREM-1 expression in and around the hepatic sinusoid in normal liver tissue (Nguye n-Lefebvre et al., 2018). Interestingly, TREM-2 expression on monocytes and macrophages was located around fibrous septa, sinusoids and areas of inflammation in human cirrhotic liver (Perugorria et al., 2019). In a recent study, an unbiased scRNA-Seq approach helped to further characterize the heterogenous population of MDMs in humans. A new pro-fibrogenic scar-associated TREM2^+^CD9^+^MNDA^+^ subpopulation of macrophages (scar-associated macrophages, SAMs) was identified, which expanded in the liver in the context of fibrosis, originated from differentiating circulating monocytes (Ramachandran et al., 2019) and was distinct from KCs (Ramachandran et al., 2020). Spatial analysis located SAMs at fibrotic niches in livers of cirrhosis patients and showed expansion of SAMs (Ramachandran et al., 2012; Ramachandran et al., 2019). SAMs also promoted HSC collagen production and proliferation (Ramachandran et al., 2019; Ramachandran et al., 2020). Strikingly, mouse injury-associated macrophages show overlap in distinct marker genes observed in human SAMs, such as TREM2 and CD9, which are conserved across species (Ramachandran et al., 2019; Xiong et al., 2019). Recent scRNA-Seq combined with cellular indexing of transcriptomes and epitopes by sequencing (CITE-Seq) and spatial proteogenomics experiments on NAFLD mouse models showed the expansion of lipid-associated macrophages (LAMs) in the liver, originating from the BM prior to the development of fibrosis and cirrhosis (Remmerie et al., 2020a; Guilliams et al., 2022). The hepatic LAMs also showed some overlap with the SAMs in expression of Trem-2 and Cd9, but could be distinctively characterised by their expression of Spp1 (Remmerie et al., 2020a).

#### 3.2.4 TAM receptor expression on macrophages

Moreover, macrophages expressing MERTK accumulated in the liver, but also the peritoneal cavity and mesenteric lymph nodes, in patients with decompensated cirrhosis and ACLF (Bernsmeier et al., 2015b). MERTK-expressing monocytes were characterised by increased expression of pro-resolution/anti-inflammatory and tissue- and lymph node-homing markers: HLA-DR^hi^CD163^hi^CX3CR1^hi^CCR7^hi^ and Ki-67 negative, which suggests an origin from recruited monocytes (Bernsmeier et al., 2015b). MERTK-expressing monocytes showed attenuated cytokine responses and an enhanced migratory potential across endothelial layers, suggesting their capacity to infiltrate the liver and reverse migrate back to the circulation where they aggravate the systemic immuneparesis (Bernsmeier et al., 2015b; Triantafyllou et al., 2018). In the context of ALF, it has been shown that these MERTK-expressing macrophages in the liver exhibited enhanced efferocytosis and played a role in resolution of inflammation and tissue restoration (Bernsmeier et al., 2015b; Triantafyllou et al., 2018). Yet, MERTK expressing cells were essential to maintaining homeostasis in the cirrhotic liver, but aggravated systemic immuneparesis (Bernsmeier et al., 2015b; Triantafyllou et al., 2018). In a mouse model for non-alcoholic steatohepatitis (NASH) it was shown that AXL signalling in primary fibroblasts, hepatocytes and liver macrophages promoted fibrosis, while GAS6 or MERTK activation protected primary hepatocytes against lipid toxicity, and the AXL inhibitor bemcentinib diminished liver inflammation and fibrosis in mice (Tutusaus et al., 2020).

#### 3.2.5 Function of macrophages in cirrhosis

The impact of aetiologies on the function of monocytes and macrophages at the stage of cirrhosis has not been extensively studied. NAFLD- and ARLD-related cirrhosis display a similar histopathology, pathophysiology, shared genetic and epigenetic factors and frequently coexist (Idalsoaga et al., 2020). Prior to the presence of cirrhosis, in ARLD and NAFLD, hepatic macrophages become activated by LPS, IFN-ɣ, and GM-CSF signalling and show pro-inflammatory cytokine/chemokine secretion (IL-6, IL-1β, and CCL2) (Mandrekar and Szabo, 2009; Gao et al., 2019; Remmerie et al., 2020b). In NAFLD, free fatty acids derived from white adipose tissue promoted hepatocyte triglyceride synthesis and storage as well as lipotoxicity with increased production of both pro-inflammatory cytokines such as TNF-α and IL-6 and macrophage recruiting chemokines such as CCL2, CCL5, and CXCL10 (Kazankov et al., 2019). NAFLD- and ARLD-related cirrhosis was associated with steatosis and hepatocyte cell death (apoptosis, necrosis and pyroptosis) promoting tissue inflammation and injury (Itoh et al., 2013; Gao et al., 2019; Miyata and Nagy, 2020). Autophagy can protect against ARLD tissue damage (Gao et al., 2019) by degrading interferon regulatory factor 1 (IRF1) and damaged mitochondria in hepatic macrophages (Ilyas et al., 2019; Liang et al., 2019; Williams and Ding, 2020). In non-cirrhotic viral hepatitis, KC activation led to the expression of IL-6, IFN-ɣ, and ROS, which in turn inhibited HCV replication and induced apoptosis of infected hepatocytes (Broering et al., 2008; Boltjes et al., 2014). In the setting of non-cirrhotic chronic HBV infection, an impaired immune response was associated with the release of IL-10 (Liu et al., 2018), reduced IL-12 expression (Wang et al., 2013) or increased expression of PD-L1 (Tian et al., 2016). However, in the context of cirrhosis, HCV also triggered the secretion of CCL5 by human macrophages and induced CCR5-dependent activation of HSCs (Sasaki et al., 2017). Patients with cirrhosis due to viral hepatitis showed increased numbers of hepatic macrophages associated with the infiltration of other pro-inflammatory CD14^+^HLA-DR^hi^CD206^+^ myeloid cells (Tan-Garcia et al., 2017), which in turn produced pro-inflammatory cytokines such as IL-1β, IL-18, and TNF-α (Weston et al., 2019).

Hepatic macrophages show tolerance during liver homeostasis and protect the liver and subsequently the systemic circulation from bacteria and bacterial products. In chronic liver disease and cirrhosis, KCs become activated and MDMs are recruited to the liver. Recently, due to new ‘omics’ technologies a list of macrophage markers associated with fibrosis, steatosis and monocyte recruitment could be identified. Hepatic macrophages show a very heterogenous phenotype expressing pro-inflammatory cytokines, but switching to a more immunoregulatory phenotype characterised by pro- and anti-inflammatory cytokines in advanced stages especially AD/ACLF. The further delineation of macrophages in relation to distinct aetiologies underlying cirrhosis and to specific stages of disease is the focus of ongoing research and the basis of potential future therapies.

### 3.3 Intestinal macrophages

Cirrhosis is a systemic disease that displays immunological alterations in immune cells beyond the liver. The gut connects to the liver by the biliary tract and portal vein, allowing for direct transfer of gut-derived components that impact liver pathophysiology (Hartmann et al., 2015) (Figure 2). In healthy conditions the intestinal mucosal and vascular barrier serve as an intersection for the interactions between the gut and the liver in order to limit systemic dissemination of microbes and toxins as well as to allow nutrients to access the circulation and liver (Albillos et al., 2020). Intestinal macrophages are a subset of CD33^+^CD14^−^ lamina propria immune cells and are hyporesponsive to LPS (Smythies et al., 2005; Smith et al., 2011). Gut-resident CX3CR1^+^ macrophages contribute to host defence and barrier integrity through their phagocytic capacity (Ber nsmeier et al., 2020). Immunosuppressive cytokine IL-10 plays a key role in the regulatory functions of intestinal macrophages (Scott and Mann, 2020).

In cirrhosis, pathological BT from the gut to mesenteric lymph nodes is driven by a suppressed intestinal immune system with small intestinal bacterial overgrowth and reduced microbial diversity (Albillos et al., 2020; Chopyk and Grakoui, 2020). Following pathological BT in patients with decompensated cirrhosis, CD14^+^TREM1^+^iNOS^+^ intestinal monocyte-derived macrophages were recruited from the circulation into the duodenum via MCP-1 secreted by intestinal epithelial cells (Du Plessis et al., 2013). Functionally these activated macrophages were responsive to microbial challenges, which resulted in the production of pro-inflammatory cytokines (IL-23, IL-6, and TNF-α) and contribute to mucosal, epithelial, vascular and immunological barrier dysfunction in cirrhosis (Kamada et al., 2008; Wiest et al., 2017; Sarin et al., 2019; De Muynck et al., 2021). If all defence mechanisms fail, these processes manifest as infection e.g., SBP, bacteraemia and sepsis (Albillos et al., 2020; Ber nsmeier et al., 2020). In murine models of cirrhosis, the mucus layer was reduced in thickness, with a loss of goblet cells, decreased MUC2 expression and bacterial overgrowth. In a NAFLD murine model recent data indicated that a high-fat diet altered the microbiome and impaired the intestinal barrier and the gut vascular barrier, which was confirmed in human gut biopsies (Mouries et al., 2019). Also chronic alcohol intake in humans leads to intestinal inflammation and increased numbers of TNF-α producing monocytes and macrophages in the lamina propria enhancing the intestinal permeability (Chen et al., 2015). It can further lead to intestinal gut microbiota dysbiosis and impaired intestinal barrier integrity (Gao et al., 2019; Sarin et al., 2019). It was shown in humans and mice that chronic alcohol use suppressed intestinal regeneration through reduced antimicrobial Reg3b and Reg3g secretion by Paneth cells (Yan et al., 2011; Hartmann et al., 2013), which led to increased bacteria adhesion to mucosal surfaces, intestinal bacterial overgrowth, enhanced BT of viable bacteria and worsening of liver inflammation (Albillos et al., 2020). Analogously, in cirrhosis patients with chronic HBV and HCV an increased gut permeability and resulting BT was observed (Sandler et al., 2011), indicating shared mechanisms in NAFLD, ARLD and viral-related cirrhosis of the liver. The mechanisms leading to increased gut permeability are not fully understood, but nitration and oxidation of tubulin, damage to microtubule cytoskeleton and activation of iNOS and NF-κB have been shown to impact tight and adherens junctions in the intestinal epithelium (Rao, 2009; Yona et al., 2013; Hartmann et al., 2015; Gao et al., 2019).

In cirrhosis, dysfunctional immune cells are not limited to the liver and the circulation but extend to the innate immune barriers of the intestine. Dysbiosis in the gut and the compromised intestinal barrier led to BT to the liver and circulation, prompting and maintaining systemic inflammation, immuneparesis and bacterial infection susceptibility.

### 3.4 Peritoneal macrophages

Under healthy conditions peritoneal macrophages (PMs), i.e. macrophages in the peritoneal cavity fluid (PF) are constantly exchanged between blood and PF and clear debris as well as pathogens (Heel and Hall, 2005) (Figure 2). In cirrhosis ascites, the accumulation of fluid in the peritoneal cavity, and SBP may develop. SBP results from intestinal and peritoneal barrier failure, which allows viable bacteria to enter the peritoneal cavity (Van der Merwe et al., 2021) and can induce AD of the liver, ACLF, multiorgan failure and death. Three subsets of PMs have been defined: Classical CD14^++^CD16^−^, intermediate CD4^++^CD16^+^ and large granular CD14^hi^CD16^hi^ (Ruiz-Alcaraz et al., 2016; Ruiz-Alcaraz et al., 2018). PMs highly express pattern recognition receptors (CD14, CD16), as well as phagocytosis receptor CD11b, cytokine receptors (CD116, CD119), T cell receptor ligand (HLA-DR), and co-stimulatory molecules (CD40, CD80) compared to circulating blood monocytes (Ruiz-Alcaraz et al., 2016; Ruiz-Alcaraz et al., 2018). Pathogens and bacterial products originating from BT from the gut may be absorbed by the peritoneal cavity and elicit an inflammatory reaction (Capobianco et al., 2017).

In regard to the aetiology, in ARLD cirrhosis PMs showed a more pro-inflammatory profile, while in hepatitis C higher levels of IL-12 and lower IL-10 levels were found (Tapia-Abellán et al., 2012). The expression of complement receptor of the immunoglobulin superfamily (CRIg) and CCR2 were found to define two phenotypically and functionally distinct peritoneal macrophage subpopulations (Irvine et al., 2016). CRIg^hi^ macrophages showed higher expression of efferocytosis receptors MERTK and TIMD4 and were on a functional level highly phagocytic and displayed enhanced antimicrobial effector activity compared to CRIg^lo^ macrophages (Irvine et al., 2016). Consequently, a high proportion of CRIg^hi^ macrophages was associated with reduced morbidity and mortality (Irvine et al., 2016). Another group defined PM subsets as large PMs (LPMs, CD206^+^CD163^+^) and small PMs (SPMs, CD206^-^), which differed in granularity and maturation markers in ascites samples from patients with cirrhosis (Stengel et al., 2020). LPMs had an inflammatory phenotype, were less susceptible to tolerance induction and released more TNF-α than SPMs. TLR stimulation and live bacteria altered levels of CD206 on the surface and resulted in release of soluble CD206 (sCD206) (Stengel et al., 2020). In the early phase of SBP, LPMs were lost, but their abundance was reversible and increased after treatment (Stengel et al., 2020). PMs co-expressed classical M1 and M2 markers illustrating their functional plasticity (Ruiz‐Alcaraz et al., 2020) and the potential of the ascitic microenvironment to dynamically alter the macrophage phenotype in order to suit the insult. In the event of BT, increased pro-inflammatory cytokine production and increased iNOS/NO production (Van der Merwe et al., 2021) and a reduced HLA-DR expression in CD14^+^ PMs was associated with adverse prognosis (Frances, 2004; Fagan et al., 2015). A counter-regulatory process resulted in ascitic IL-10, resin and reduced expression of CD14, CD16, HLA-DR, CD86, and CD206 on PMs (Lesińska et al., 2014; Nieto et al., 2015; Nieto et al., 2018; Hadjivasilis et al., 2021). The reduced CD14 expression on PMs in SBP conditions was associated with impaired phagocytosis (Nieto et al., 2015; Nieto et al., 2018). When comparing peripheral circulating monocytes in stable cirrhosis conditions to PMs of patient with ACLF increased immunoregulatory MERTK-expression population was found, indicating a more suppressive phenotype of PMs in ACLF patients (Bernsmeier et al., 2015b; Flint et al., 2022).

Taken together, in the peritoneal fluid from patients with decompensated cirrhosis complicated by ascites, two distinct PM populations have been identified. LPMs have a pro-inflammatory phenotype (CD14^+^CD206^+^CCR2^−^CRIg^+^MERTK^+^), are less susceptible to tolerance induction, and release more pro-inflammatory cytokines (TNF-α) compared to SPMs. In SBP conditions LPMs release sCD206 and the loss of LPMs occurs, while the number of SPMs (CD14^+^CD206^−^CCR2^+/−^MERTK^-^) increases. Due to the technical challenge to date, PM from patients with compensated cirrhosis without ascitic fluid have not been characterised phenotypically and functionally. This comparison may help to identify immunological and barrier switches in the progression from compensated to decompensated stages of cirrhosis, since ascites is considered as the hallmark of decompensation (D’Amico et al., 2022).

### 3.5 Macrophages in other tissues

As a systemic disorder, cirrhosis can involve a number of other organs beyond the circulation, liver, gut and peritoneum.

Lymphoid organs like the spleen and lymph nodes harbour mononuclear phagocytes among other immune cells, which are also be affected in cirrhosis patients. In cirrhosis defective immune cells in mesenteric lymph nodes facilitate BT in decompensation stage. A study showed the accumulation of immune-suppressive MERTK-expressing macrophages in the subcapsular sinus and medullary cord in the lymph nodes of patients with AD/ACLF (Bernsmeier et al., 2015b). In the spleen of patients with cirrhosis and PH phagocytosis activity of macrophages in the red pulp and marginal zone was enhanced and related to hypersplenism and cytopenia (Yongxiang et al., 2002). There is evidence also for a spleen-liver crosstalk, given a study showed reduced fibrosis, monocyte infiltration within the injured liver and CCL2 secretion by hepatic macrophages in cirrhosis patients that had undergone splenectomy (Li et al., 2018).

Adipose tissue macrophages (ATMs) have been intensively investigated over the last years in mouse models or in patients with obesity and NAFLD, however not in the context of cirrhosis. In obesity, the adipose tissue mechanism for lipid containment fails leading to dyslipidemia and insulin resistance (Korf et al., 2019b; Remmerie et al., 2020a). Dying adipocytes release DAMPs and cause adipose tissue macrophage (ATM) activation, which form crown like structures around necrotic adipocytes to engulf cell debris (Korf et al., 2019b; Remmerie et al., 2020a). ATMs secrete pro-inflammatory cytokines (TNF-α, IL-6, and IL-1β), promote monocyte recruitment via MCP-1, increase insulin resistance, hepatic lipid flux and lipid accumulation and are associated with disease progression of NAFLD (Du Plessis et al., 2015; Lefere and Tacke, 2019). The CCL2-CCR2 and CCL5-CCR5 chemokine signalling axes stimulates monocyte recruitment to insulted adipose tissue sites and has been suggested as therapeutic target to resolve inflammation in NAFLD (Kazankov et al., 2019).

Overall, the study of macrophages in the context of cirrhosis in organs other than the liver, circulation or peritoneum remain underrepresented. This may be a result of the difficulty to obtain these biological materials such as lymph nodes, spleen, adipose and other tissue from patients. There is a substantial need to investigate immune processes systematically in diverse tissues in order to understand the plasticity of monocytes and macrophages in the human body in the context of cirrhosis.

## 4 Potential monocyte and macrophage derived markers of immuneparesis in cirrhosis

Immuneparesis in patients with cirrhosis develops as a consequence of persisting systemic inflammation, which attenuates the responses of immune cells required to combat infections (Bonnel et al., 2011; Bajaj et al., 2012; Albillos et al., 2014). Monocyte and macrophage plasticity in tissues and the systemic circulation play a key role in the regulation of tissue homeostasis and defence against infection. Therefore, future prognostic markers indicating the state of immuneparesis would be helpful in clinical decision making.

The soluble form of two scavenger receptors CD163 and CD206 (sCD163 and sMR/sCD206), expressed by circulating blood monocytes and macrophages, were present in plasma, ascites and other body fluids (Grønbæk et al., 2020; Nielsen et al., 2020) and associated with severity and prognosis of chronic liver diseases (Grønbæk et al., 2016; Kazankov et al., 2016; Rainer et al., 2018; Grønbæk et al., 2020; Nielsen et al., 2020; Stengel et al., 2020) and cirrhosis (Holland-Fischer et al., 2011; Grønbaek et al., 2012; Rode et al., 2013; Waidmann et al., 2013). High sCD163 levels were additionally associated with variceal bleeding, but also predicted mortality in alcoholic hepatitis (Waidmann et al., 2013; Saha et al., 2019). However, sCD163 and sMR plasma/serum levels have been associated with a variety of other diseases and infection conditions (Andersen et al., 2018; Suzuki et al., 2018; Gong et al., 2021; Arneth and Kraus, 2022; Davidsson et al., 2022) as shedding of CD163 and CD206 is associated with macrophage activation (Grønbæk et al., 2020; Nielsen et al., 2020) in general and does not specifically indicate immuneparesis in patients with cirrhosis. Moreover, sCD163 plasma levels differed in relation to the underlying aetiologies (Wang et al., 2015) further mitigating their clinical significance. Given their secretion by LPM, sCD163, and sMR may potentially enhance the diagnostic criteria for SBP in ascitic fluid of cirrhosis patients (Stengel et al., 2020).

AXL expressing immune-regulatory monocytes have been shown to increase in patients during progression of cirrhosis independent of the aetiology and prior to AD and related infectious complications, as well as the future onset of AD and mortality (Brenig et al., 2020). Therefore, the flow cytometry-based analysis of AXL expression on monocytes in patients with cirrhosis might serve as a marker of immuneparesis and identify patients at risk for infection and deterioration. Also its soluble form sAXL was increased in the plasma of cirrhosis patients correlating with cirrhosis progression (Brenig et al., 2020). However, sAXL has been suggested not only as a marker for advanced fibrosis/cirrhosis but also HCC development (Dengler et al., 2017; Staufer et al., 2017). Elevated sAXL levels were not detected in patients with chronic liver diseases of diverse underlying aetiologies, yet patients with advanced fibrosis (F3), cirrhosis (F4) (54.67–94.74 ng/ml) and HCC (82.7–114.5 ng/ml) displayed higher levels compared to healthy controls (40.15 ng/ml) (Dengler et al., 2017). By contrast, MERTK expression has been identified as a marker both for immunosuppressive circulating monocytes and for macrophages in the liver, peritoneum and mesenteric lymph nodes in acute decompensation of cirrhosis (AD/ACLF) (Bernsmeier et al., 2015b). Thus, in addition to AXL expression, flow cytometry-based MERTK expression on monocytes may represent a valuable marker of immuneparesis and indicating patients with AD/ACLF.

The progression from compensated to decompensated stages of cirrhosis is associated with a poor prognosis and a high mortality (D’Amico et al., 2022). Given the increase in circulating neutrophils and concomitant decrease in lymphocyte numbers is a response to stress including sepsis (de Jager et al., 2010), the neutrophil-lymphocyte ratio (NLR) and monocyte-lymphocyte ratio (MLR) have been developed as prognostic markers for patient stratification. Patients with AD and ACLF who died during hospitalisation displayed elevated NLR and MLR (Bernsmeier et al., 2020). In ACLF patients, an NLR >30 was associated with an 80% 3-months mortality risk (Bernsmeier et al., 2020). Thus, NLR and MLR may be used as rapidly available robust initial diagnostic tools for patient stratification in AD/ACLF.

## 5 Therapeutic modulation of monocyte and macrophage function in immuneparesis

Along cirrhosis progression patients develop multiple complications (PH, inflammation, BT, gut dysbiosis) that predispose them to decompensation and subsequent precipitating events (infection, variceal haemorrhage) lead to AD (Gustot et al., 2021).

The association of immuneparesis with infectious complications, decompensation and mortality has evoked the concept of immunomodulatory therapies as an adjuvant concept in order to prevent decompensation, save organs and reduce mortality in patients with cirrhosis. Over the previous decade, several potential targets for immunotherapy have been defined (Table 1).

**TABLE 1:** Potential immunomodulatory therapies targeting monocyte/macrophages in cirrhosis.

Target	Compound	Mechanism	References
CD34+ hematopoietic stem cells	G-CSF	G-CSF promotes release of CD34+ hematopoietic stem cells from the bone marrow which may differentiate into monocytes and replace dysfunctional subsets	Kedarisetty et al. (2015)
			Verma et al. (2018)
			Newsome et al. (2018)
			Engelmann et al. (2021)
TLR-4	TAK-242 or SERELAXIN	Inhibition of macrophage TLR-4 by TAK-242 or SERELAXIN reduces liver injury and inflammation in rodent models	Shah et al. (2013)
			Engelmann et al. (2020)
			Bennet et al. (2017)
NLRP3	MCC950	NLRP3 inflammasome inhibition by MCC950 reduces liver inflammation and fibrosis in experimental models of NASH	Mridha et al. (2017)
MERTK	UNC569	MERTK inhibition restored LPS-induced pro-inflammatory cytokine response in monocytes from patients with ACLF *ex vivo*	Bernsmeier *et al.* (2015b)
AXL	BGB324	AXL inhibition restored LPS-induced pro-inflammatory cytokine response in monocytes from patients with cirrhosis *ex vivo*	Brenig et al. (2020)
M-MDSC	Poly(I:C)	TLR-3 agonism by Poly(I:C) reversed M-MDSC expansion and increased the antimicrobial function of these cells in patients with ACLF *in vitro*	Bernsmeier et al. (2018)
Glutamine Synthetase	Methionine sulfoximine	Inhibition of glutamine synthetase partially restored both phagocytosis and inflammatory response of monocytes conditioned with plasma from patients with ACLF *in vitro*	Korf et al. (2019a)
Prostaglandin E2	Albumin	Albumin administration in patients with ESLD lowered circulating PGE2 levels and restored TNF-α production by plasma-conditioned MoMF *in vitro*	O’Brien et al. (2014)
			China et al. (2018)
CCR2/CCR5	Cenicriviroc	Therapeutic treatment with Cenicriviroc reduced recruitment of hepatic Ly-6C+ MoMF in experimental mouse models of NASH	Krenkel et al. (2018)

G-CSF; granulocyte colony-stimulating factor; TLR-4; Toll-like receptor 4; NLRP3; NOD-, LRR- and pyrin domain-containing protein 3; NASH; Non-alcoholic steatohepatitis; MERTK; myeloid-epithelial-reproductive tyrosine kinase; LPS; Lipopolysaccharide; ACLF; Acute-on-chronic liver failure; AXL; Anexelekto; M-MDSC; Monocytic myeloid-derived suppressor cells; Poly(I:C); Polyinosinic-polycytidylic acid; TLR-3; Toll-like receptor 3; ESLD; End-stage liver disease; TNF-α; Tumor necrosis factor α; PGE2; Prostaglandin E2; MoMF; Monocyte-derived macrophages; CCR2/CCR5; C-C chemokine receptor type 2/5.

Firstly, therapies with G-CSF have gained interest. These therapies aim to improve liver regeneration and innate immune responses in cirrhosis through the release of bone marrow-derived CD34^+^ haematopoietic stem cells in order to replace the dysfunctional circulating monocytes and have been studied intensively in ACLF patients (Kedarisetty et al., 2015; Newsome et al., 2018; Verma et al., 2018). However, distinct clinical studies showed differing outcomes. On one hand G-CSF improved the survival of patients (Garg et al., 2012; Verma et al., 2018), while on the other hand G-CSF with haemopoietic stem-cell infusion did not improve liver dysfunction and was associated with increased frequency of adverse events (Newsome et al., 2018). Most recently, a large prospective multicentre trial failed to show beneficial effects of G-CSF over standard of care alone (Engelmann et al., 2021). Thus, G-CSF treatment cannot be recommended at any stage of cirrhosis to date and may require further detailed investigation.

Another therapeutic approach may involve inhibition of macrophage activation via pattern recognition receptors by TLR4 inhibition using TAK-242 or SERELAXIN. Proof of concept studies of TLR4 inhibition previously revealed reduced liver injury and inflammation in rodent models (Seki et al., 2007; Shah et al., 2013; Bennett et al., 2017; Engelmann et al., 2020). In a similar fashion the NLRP3 inflammasome inhibitor MCC950 reduced liver inflammation and fibrosis in experimental NAFLD models (Mridha et al., 2017). These concepts await further evaluation in human *in vitro* models and clinical studies.

*Ex vivo* data obtained from patients at different stages of cirrhosis support the potential strategy of targeting TAM receptors AXL and MERTK (Bernsmeier et al., 2015b; Brenig et al., 2020). The TAM receptor inhibitors UNC569 (MERTK) and BGB324 (AXL) augmented pro-inflammatory cytokine response in *ex vivo* models (Bernsmeier et al., 2015b; Brenig et al., 2020) and therefore deserve evaluation in *vivo* models of cirrhosis and ACLF. TAM receptor inhibitors are in clinical evaluation (phase 2 studies) for different malignant diseases and may eventually be translated to liver disease given their known safety profiles. Another candidate is the established diabetes drug metformin, which led to reduced AXL expression on monocytes and enhanced inflammatory cytokine responses *in vitro* (Brenig et al., 2020). Moreover, the use of metformin in patients with cirrhosis and diabetes appeared safe and was associated with reduced mortality, HCC or decompensation (Kaplan et al., 2021).

Another candidate for immunotherapy may be the TLR3 agonist poly (I:C), which reduced proportions of M-MDSCs and improved their anti-microbial function *in vitro* (Bernsmeier et al., 2018). Poly (I:C) has been used as an adjuvant for vaccination and thus *in vivo* studies for the treatment of cirrhosis may be considered.

In addition, feeding of glutamine into the tricarboxylic acid cycle by using a pharmacological inhibitor of glutamine synthetase (GLUL) restored innate immune responses, in particular the phagocytic and inflammatory capacities of monocytes from patients with ACLF (Korf et al., 2019a).

The therapeutic effects of inhibiting monocyte infiltration by using cenicriviroc (CVC), an oral dual chemokine receptor CCR2/CCR5 antagonist, were assessed using mouse models (Krenkel et al., 2018). In all murine models for steatohepatitis, as well as liver fibrosis progression and fibrosis regression, CVC treatment reduced the recruitment of MDMs (Krenkel et al., 2018). Furthermore, CVC did not affect macrophage polarization, hepatocyte fatty acid metabolism or HSC activation (Krenkel et al., 2018). The inhibition of CCR2^+^ monocyte recruitment shows potential to improve fibrosis in patients with NASH (Krenkel et al., 2018), but needs further evaluation for the potential of cirrhosis reversibility and its effect on cirrhosis due to other underlying liver diseases.

PAMP-mediated macrophage activation may also be prevented by the restoration of a normal gut microbiome using antibiotics and probiotics (Mazagova et al., 2015; Irvine et al., 2019; Wen et al., 2021). Novel approaches to antibiotic prophylaxis have been explored, including strategies targeting intestinal dysbiosis, including non-selective decontamination (rifaximin), probiotics and faecal microbiota transplantation. Moreover, intestinal motility and barrier function may be improved by the use of prokinetics, beta-blockers, and bile acids (Yan and Garcia-Tsao, 2016). Treatments improving intestinal homeostasis could beneficially impact systemic immune function by reducing exposure to pathological taxa and chronic immune stimulation. Many antibiotics, including those prophylactically used in patients with decompensated cirrhosis, have direct impact on the immune system, although the underlying mechanisms are not well-defined (Zapater et al., 2015).

In addition, the cyclooxygenase (COX)-derived eicosanoid prostaglandin E_2_ (PGE_2_) was elevated in the plasma of decompensated cirrhosis patients in association with suppressed cytokine responses and bacterial killing by macrophages *in vitro* (O'Brien et al., 2014). Albumin and COX inhibitors modulated endosomal TLR signalling and improved pro-inflammatory cytokine production (O'Brien et al., 2014; China et al., 2018; Casulleras et al., 2020). An *in vitro* study revealed that the augmentation of serum albumin above 30 g/L was associated with the reversibility of plasma-mediated immune dysfunction by binding and inactivation PGE_2_ (China et al., 2018). Intravenous albumin had however no effect on systemic inflammation, albumin function, cardiovascular mediators and biomarkers compared with standard of care in hospitalized decompensated cirrhosis (China et al., 2022). To date, the standard of care remains the treatment with albumin in addition to appropriate antibiotics in SBP (Bajaj et al., 2021) as well as in acute kidney injury. Further studies may evaluate the additive use of COX inhibitors and their potential to improve immuneparesis and restore cytokine production in cirrhosis patients.

In summary, there are a variety of immunomodulatory strategies in clinical evaluation which may shape the future therapy of patients with cirrhosis by improving their antimicrobial defence, and hereby hopefully save organs and reduce mortality.

## 6 Conclusion

In conclusion, research over the last decade has significantly improved our understanding of the plasticity of monocyte and macrophage differentiation in relation to different compartments and clinical conditions. Their relevance in immuneparesis development in patients with cirrhosis as well as their prognostic value is substantial, and thus supports the concept of adjuvant immune-modulatory treatments. This review specifically summarizes findings originating from patient data or distinctly describing the translation from mouse to human findings. To date immune-pathophysiological findings in humans with cirrhosis are little due to the limitation in assessing samples. Furthermore, translation of mouse related findings to human have to be drawn carefully due to the lack of a proper cirrhosis mouse model. Methodological advances such as single-cell based technologies have enabled the identification of tissue specific mononuclear phagocytic subsets in the context of their functions in limited samples from patients with liver cirrhosis. However, further studies are needed to clarify monocyte and macrophage function also in relation to the diverse underlying aetiologies and in diverse compartments other than the liver and the circulation. Decompensation of cirrhosis is accompanied with the development of diverse complications, such as ascites and infections. Infections, which are due to a dysfunctional immune response, in turn represent a great risk to patients with decompensated cirrhosis. Most of these infections originate from barrier dysfunction in the gut and peritoneum, which result in BT to the circulation. In addition to the inflamed liver, BT has the potential to change the milieu in the gut, liver, circulation and peritoneum and affects tissue specific differentiation of monocytes and macrophages. Treatment with antibiotics and albumin have reduced the mortality in case of infection, but antibiotics should be prescribed with caution given the increasing prevalence of MDRO in cirrhosis patients. Research over the past years has identified not only potential prognostic molecular markers to better predict cirrhosis progression and immuneparesis development in patients, but also molecules on immune cells which may be targeted in order to restore immune function and prevent infectious episodes. The development of immune-modulatory treatments however is challenging given the tissue-specific plasticity of immune cells and requires careful evaluation *in vivo*. At present, experimental studies and clinical trials aimed at either identifying novel immunotherapeutic targets or delineating beneficial effects of distinct immune-modulatory treatments from their off-target effects are ongoing.
